# Long-term follow-up of implants placed after Le Fort I osteotomy with interpositional autogenous bone grafts: a retrospective study

**DOI:** 10.1186/s40729-025-00661-3

**Published:** 2025-12-08

**Authors:** H. Persson, C. Östlund, L. Rasmusson, J. Walladbegi

**Affiliations:** Department of Oral and Maxillofacial Surgery, Institute of Odontology, The Sahlgrenska Academy, University of Gothenburg, Gothenburg, Sweden

**Keywords:** Edentulous, Le Fort I, Osteotomy, Oral implants, Osseointegration

## Abstract

**Background:**

Oral implantology has become a standard procedure for replacing missing teeth. However, patients with severe maxillary bone resorption often require complex surgical interventions, such as Le Fort I osteotomy with interpositional bone grafting, prior to implant placement. This study aimed to evaluate the long-term outcomes of oral implants placed following this surgical technique.

**Patients and methods:**

The present study was two-fold. First, a retrospective review was performed using medical records and conventional panoramic radiographs from 26 patients who had undergone Le Fort I osteotomy with interpositional bone grafting and received either Astra Tech or Brånemark oral implants. Patients were followed for up to 5 years. Marginal bone loss was measured from the implant shoulder to the bone level. Implant survival and success rates were assessed, with success defined as bone loss of ≤ 2 mm during the first year and < 0.2 mm annually thereafter. Second, to assess the long-term outcomes of the two oral implant systems, all traceable patients were invited to undergo a follow-up radiographic examination up to 29 years post-treatment.

**Results:**

The combined survival rates for Astra Tech and Brånemark oral implants were 97.0%, 94.6%, and 94.1% at 1, 2, and 3 years, respectively, with no additional implant loss observed by year 5. The overall success rate at 5 years was 56.1%, with Astra Tech implants showing a higher success rate (70.8%) compared to Brånemark implants (51.4%). Although there was no statistically significant difference in survival rates between the two systems, Astra Tech implants demonstrated significantly less marginal bone loss at both 1-year (*p* < 0.01) and 3-year (*p* = 0.021) follow-ups. For the long-term evaluation, 14 patients (54%) from the original cohort were traceable, of whom 4 patients (29%) participated in the follow-up. Among the 28 Brånemark implants assessed, none were lost, resulting in a 100% survival rate up to 29 years. The mean marginal bone loss of 2.6 ± 1.8 mm.

**Conclusion:**

Le Fort I osteotomy with interpositional bone grafting, followed by oral implant placement and prosthetic rehabilitation, appears to be a reliable long-term treatment option for patients with severe maxillary atrophy, demonstrating favorable long-term survival-, and success rates.

## Introduction

Over the past decades, oral implant treatment has evolved into a standard clinical procedure for replacing missing teeth and restoring both function and aesthetics for patients [1].

While a variety of implant materials are available, titanium remains the most extensively documented and widely used [2]. Currently, approximately 200 implant brands are produced by around 80 manufacturers worldwide, offering a wide range of implant systems with varying lengths, diameters, and surface modifications, both mechanical and chemical [3]. This diversity allows for personalized treatment planning tailored to individual patient needs, particularly in complex cases involving compromised bone volume.

Historically, when oral implant designs were limited in length and diameter, treating patients with severe bone resorption posed significant challenges. To address these limitations and achieve adequate bone volume for implant placement, various surgical interventions were introduced. In cases of severely resorbed edentulous maxillae, Le Fort I osteotomy with interpositional bone grafting emerged as an effective solution. However, this approach is resource-intensive, typically requiring general anesthesia, extended hospitalization, and carrying a risk of post-operative complications [4, 5].

Today, Le Fort I osteotomy remains a fundamental procedure for managing skeletal maxillofacial deformities. Briefly, the technique involves an intraoral surgical cut is made above the upper teeth, allowing the maxilla to be separated from the skull base while preserving its vascular supply. The maxilla is then repositioned and stabilized using plates and screws [4]. When additional bone volume is required, autogenous bone, typically harvested from the iliac crest, is grafted interpositionally between the maxilla and the skull base [4]. Oral implants may be placed either immediately during the osteotomy (one-stage procedure) or in a subsequent surgical session (two-stage procedure), followed by prosthetic rehabilitation [5, 6].

Advancements in implantology, such as the introduction of short implants, have significantly reduced the need for extensive graft procedures. For instance, a recent study demonstrated favorable long-term outcomes in fully edentulous patients rehabilitated with implant-supported restorations, achieved without the need for bone grafting procedures [7]. However, in the present study, the indication for treatment extended beyond insufficient bone volume. A clinically significant sagittal discrepancy between the jaws, resulting from severe bone resorption, also necessitated surgical correction.

Understanding the survival-, and success rates of implants placed in conjunction with this technique provides valuable insight into long-term outcomes and informs clinical decision-making in cases where conventional implant therapy is not feasible. Accordingly, the aim of this study was to evaluate the outcomes of oral implants placed following Le Fort I osteotomy with interpositional autogenous bone grafting.

## Patients and methods

### Study design

This study was two-fold. First, a retrospective review of medical records and conventional panoramic radiographs was performed for patients who had been enrolled in one of two previous studies [5, 8]. These patients were edentulous and presented severely resorbed maxillae. Based on their eligibility for Le Fort I osteotomy with interpositional bone grafting, they were randomized to receive one of two oral implant systems (Astra Tech or Brånemark). Following surgery, all patients underwent full-arch oral implant placement and were subsequently restored with a fixed prosthesis. Second, to evaluate the long-term outcomes of the two oral implant systems, all patients who could be traced at the time of the present study were invited to undergo an additional radiographic examination.

All available medical records and conventional panoramic radiographs from the original studies were reviewed. The inclusion criteria for surgical treatment was a completely edentulous maxilla with a residual vertical bone height of 5 mm or less. The exclusion criteria were as follows: (i) follow-up period ≤ 2 years; (ii) < 2 panoramic radiographs during the follow-up period; (iii) additional surgical procedures beyond Le Fort I osteotomy with interpositional bone grafting; and (iv) < 6 implants placed. Historical data were retrieved from the Regional Archive in Västra Götaland and analyzed at the Department of Oral and Maxillofacial Surgery, a regional center for orthognathic surgery at Sahlgrenska University Hospital, Gothenburg, Sweden. The review of medical records and panoramic radiographs was conducted between August 16 and November 30, 2022, by two calibrated investigators (HP and CÖ) to ensure consistent data extraction, minimize missing data, and maintain standardization throughout the study.

### Procedures and data collection

All panoramic radiographs, both conventional and digital, were assessed for image quality to identify potential sources of error. Patient positioning was evaluated with respect to tongue placement, chin position, and cervical spine alignment, distance from the detector and head centering. Image quality was further assessed based on exposure and the clarity of the bone level. In cases where the bone level was difficult to discern, a senior maxillofacial radiologist was consulted.

Conventional panoramic radiographs were reviewed using a lightbox (Molanders, MOD QUP/A3SL) and a stainless-steel ruler with a 0.5 mm scale. Bone loss was measured according to a previously published method [9], by calculating the distance from the implant shoulder to the marginal bone level on both the mesial and distal surfaces of each implant. Baseline bone level was defined as the marginal bone-to-implant contact observed on the initial radiograph taken at the time of implant placement, with 0 mm representing no bone loss. Given that the magnification factor of conventional radiographs ranged between 1.3 × and 1.7 × , measurements were adjusted accordingly by dividing the measured values by 1.3 or 1.7. The corrected values were expressed in millimeters. Additionally, the number of missing or lost implants was recorded. For digital panoramic radiographs, the Planmeca ProMax 3D system (Planmeca OY, Helsinki, Finland) was used. All digital images were stored and analyzed using the Sectra Workstation IDS7 version 24.1.10.5437 (Sectra AB, Linköping, Sweden), a digital imaging and documentation system commonly used in Swedish healthcare. Assessment of mesial and distal marginal bone levels, as well as implant loss, followed the same methodology as for conventional radiographs. All patients who participated in the long-term follow-up received a radiation dose of approximately 0.019 mSv. The collected data were used for statistical analyses to evaluate and compare the survival-, and success rates of the Astra Tech and Brånemark implant systems.

Implant survival was defined as the presence of implants in situ throughout the follow-up period. Success was defined according to criteria from two previously published studies [10, 11], with success classified as ≤ 2 mm of bone loss during the first year after baseline radiographs; and < 0.2 mm of additional bone loss per year thereafter. Analyses were conducted at both the implant-, and patient-levels. At the implant-level, if bone level measurements could not be obtained from one of the two surfaces (mesial or distal) the value from the contralateral surface was used. If bone levels could not be assessed on either surface, the implant was excluded from success-rate analysis for that specific year. The same approach was applied in the patient-level analysis.

### Endpoints

The primary endpoint was:

1. To compare the annual survival rates of Astra Tech and Brånemark implants up to 5 years following implant placement.

The secondary endpoints were:

- To compare the success rates of Astra Tech and Brånemark implants at 1-, 3- and 5-years following implant placement.
- To evaluate and compare the long-term survival-, and success rates of Astra Tech and Brånemark implants, approximately 30 years after placement.

### Statistical analyses

Descriptive data are presented as means ± standard deviations. The primary endpoint, i.e., cumulative incidence of survival, was assessed using Kaplan–Meier survival analysis, and end of study, death or unavailable records was used to censor follow-up time. The secondary endpoint (2a), comparing success rates at 1-, 3-, and 5-years, was analyzed using Pearson’s Chi-square test. The secondary endpoint (2b), evaluating long-term survival and success, was reported descriptively. A *p*-value ≤ 0.05 was considered statistically significant. All statistical analyses were performed using IBM® SPSS® Statistics (version 29; IBM Corp., Armonk, NY, USA).

## Results

A total of 47 patients received oral implants following Le Fort I osteotomy with interpositional bone grafting, accounting for 357 implants in total (176 Astra Tech implants [A]; 181 Brånemark system implants [B]). However, complete medical records and conventional panoramic radiographs were available for only 28 of these patients. Data for the remaining 19 patients were either incomplete or unavailable. Following an initial assessment, 2 additional patients were excluded, 1 due to missing panoramic radiographs and 1 due to an insufficient number of implants, yielding complete datasets for 26 patients, comprising a total of 202 oral implants. In total, 102 conventional panoramic radiographs were analyzed for the retrospective portion of the study. Patient characteristics are summarized in Table 1.

**Table 1:** Patient characteristics and implant details for Astra Tech and Brånemark implant systems

Implant system	Number of patients	Sex (F:M)	Mean age	Number of implants
Astra Tech	11	8:3	63 (48–73)	88
Brånemark	15	11:4	59 (39–70)	114
Combined	26	19:7	61 (39–73)	202

Patient characteristics, including implant system, number of patients, sex distribution, mean age at the time of oral implant placement, age range (in years), and total number of implants. Data are presented separately for the Astra Tech and Brånemark oral implant systems, as well as combined for both systems

**Fig. 1:**
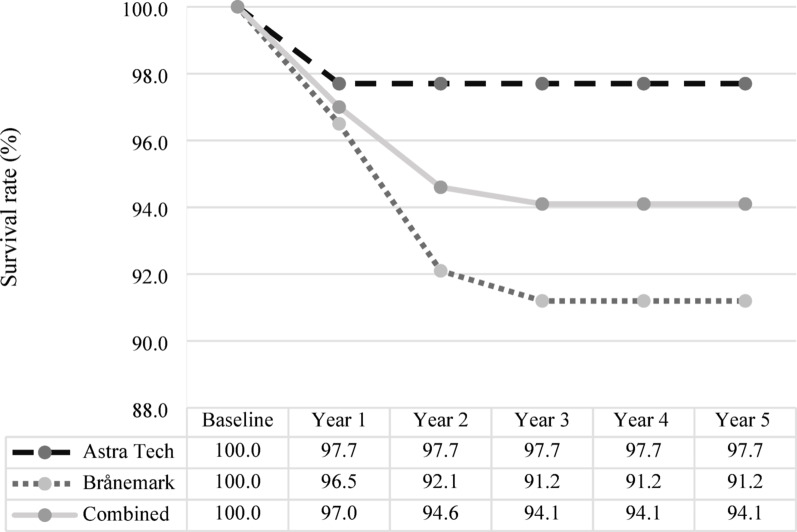
Implant-level survival rates over 5 years for Astra Tech and Brånemark implant systems. Implant-level survival rates (%) for the Astra Tech and Brånemark oral implant systems, presented separately and combined. Data are presented for baseline and annually over a 5-year period following implant placement

The primary endpoint, implant-level survival rates for the combined implant systems [A] and [B], was as follows: year 1 [A + B = 196/202; 97.0%], year 2 [A + B = 191/202; 94.6%], and year 3 [A + B = 190/202; 94.1%]. No additional implant losses were recorded during years 4 and 5, and survival rates remained unchanged from year 3 (Fig. 1, Table 2). When analyzed separately, the survival rates were as follows: year 1 [A = 86/88; 97.7%] versus [B = 110/114; 96.5%], year 2 [A = 86/88; 97.7%] versus [B = 105/114; 92.1%], and year 3 [A = 86/88; 97.7%] versus [B = 104/114; 91.2%]. No statistically significant differences in implant survival rates were observed between the two implant systems at any follow-up time point.

**Table 2:** Implant retention over 5-years for Astra Tech and Brånemark implant systems

Implant system	Baseline	Year 1	Year 2	Year 3	Year 4	Year 5
Astra Tech	88	86	86	86	86	86
Brånemark	114	110	105	104	104	104
Combined	202	196	191	190	190	190

Number of remaining implants for the Astra Tech and Brånemark oral implant systems at baseline and annually over a 5-year period following implant placement. Data are presented for each system, as well as combined

**Fig. 2:**
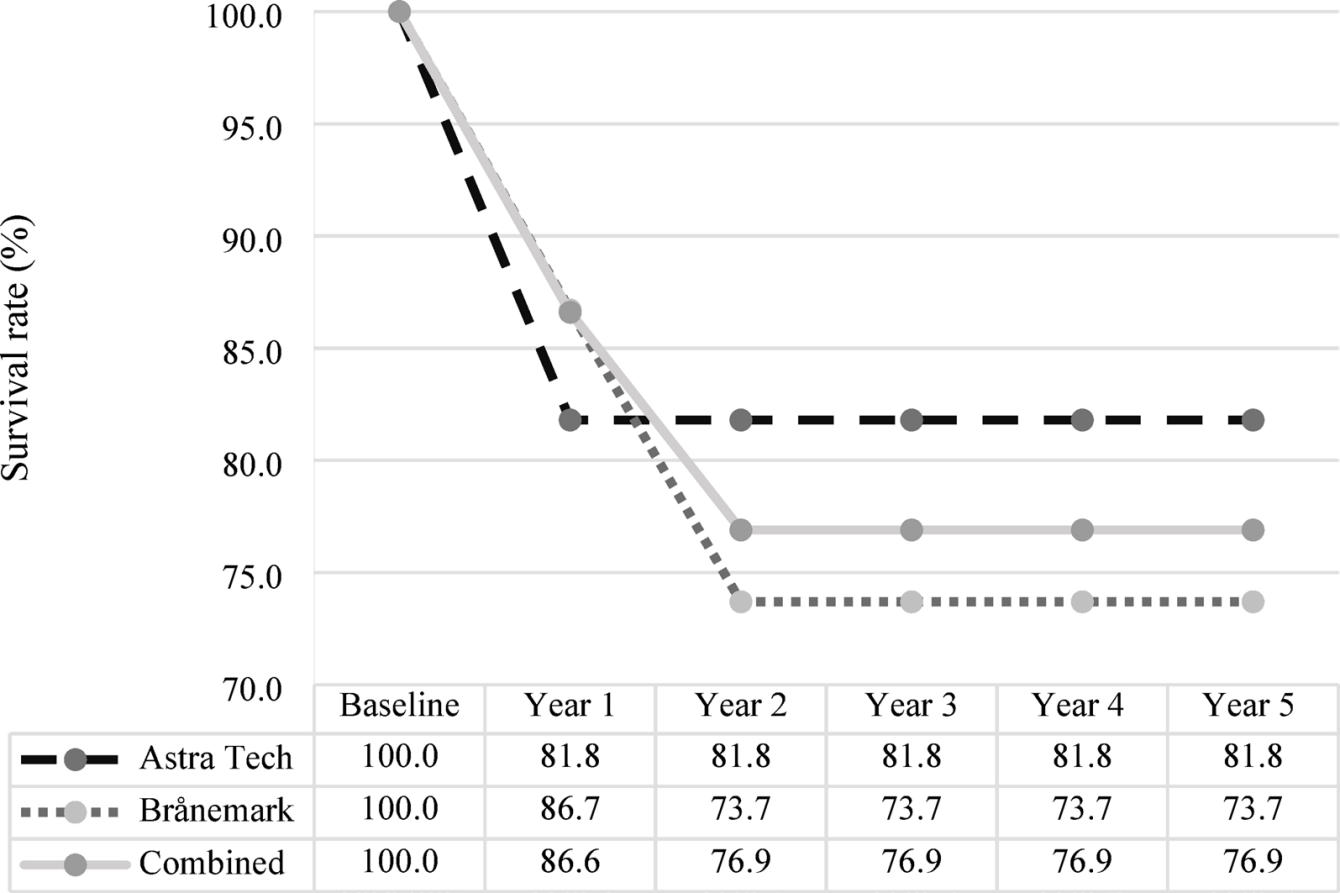
Patient-level survival rates over 5 years for Astra Tech and Brånemark implant systems. Patient-level survival rates (%) for the Astra Tech and Brånemark oral implant systems, presented separately and combined. Data are presented for baseline and annually over a 5-year period following implant placement

At the patient-level, implant loss occurred in 4 patients after year 1: [A = 2] and [B = 2]. In year 2, two additional patients experienced implant loss, both of whom had Brånemark implants [A = 0] and [B = 2]. In year 3, one of these Brånemark patients lost an additional implant [A = 0] and [B = 0]. However, this did not affect the overall patient-level outcome. No further implant losses were reported in years 4 or 5. By year 3, the cumulative patient-level survival rate for both implant systems combined was 76.9% (n = 20/26), with system-specific survival rates of 81.8% (n = 9/11) for [A] and 73.7% (n = 11/15) for [B]. This difference of approximately eight percentage points was not statistically significant (Fig. 2).

**Fig. 3:**
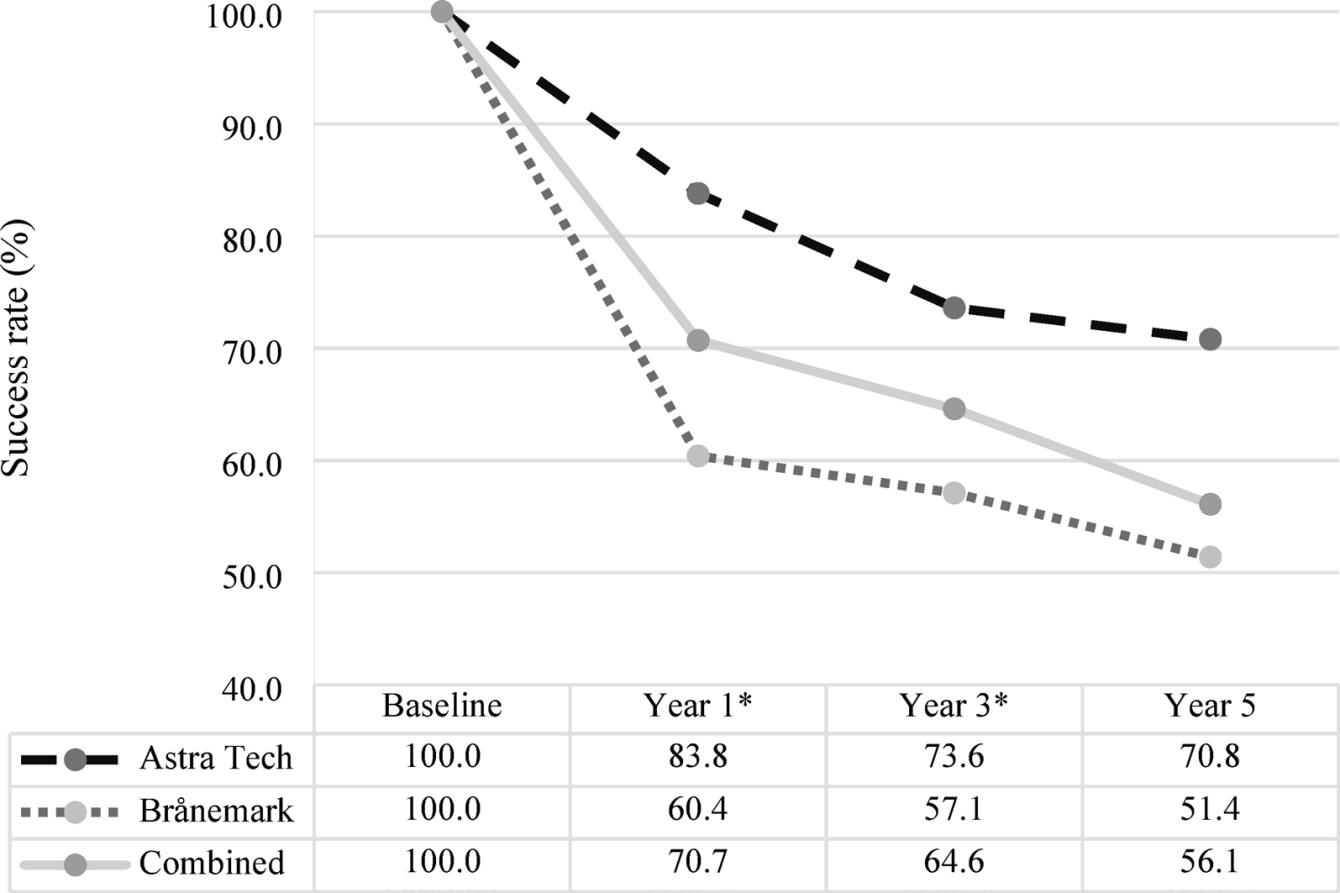
Implant-level success rates over time for Astra Tech and Brånemark implant systems. Implant-level success rates (%) for the Astra Tech [A] and Brånemark [B] oral implant systems, presented separately and combined, at baseline and at 1-, 3-, and 5-years following implant placement. An asterisk (*) indicates a statistically significant difference between systems [A] and [B]

For the secondary endpoint (2a), i.e., the implant-level success rates, 70.7% (128/181) of implants met the defined success criteria 1 year after placement when both implant systems were combined. When analyzed separately, success rates were: [A = 67/80; 83.8%] and [B = 61/101; 60.4%]. At 3 years after implant placement, the overall success rate was 64.6% (115/178), with system-specific rates of [A = 59/80; 73.6%] and [B = 56/98; 57.1%]. By 5 years, 56.1% (55/98) of implants continued to meet the success criteria. Subgroup analysis revealed success rates of [A = 17/24; 70.8%] and [B = 38/74; 51.4%]. The corresponding marginal bone loss for the combined systems was 1.1 ± 1.3 mm at year 1; 1.7 ± 1.5 mm at year 3; and 2.2 ± 1.8 mm at year 5. When analyzed by implant system, mean bone loss was as follows: year 1 [A = 0.9 ± 1.5] versus [B = 1.3 ± 1.1]; year 3 [A = 1.5 ± 1.5] versus [B = 1.9 ± 1.6]; and year 5 [A = 1.8 ± 1.9] versus [B = 2.3 ± 1.7]. Statistically significant differences in marginal bone loss between the two implant systems were observed at the 1-year (*p* < 0.01) and 3-year (*p* = 0.021) follow-ups. However, no significant difference was found at the 5-year follow-up (Fig. 3, Table 3).

**Table 3:** Implant-level success rates for the Astra Tech and Brånemark implant systems

Implant system	Year 1 (*p* < 0.01)	Year 3 (*p* = 0.021)	Year 5 (*p* = 0.095)
Astra Tech	0.9 ± 1.5	1.5 ± 1.5	1.8 ± 1.9
Brånemark	1.3 ± 1.1	1.9 ± 1.6	2.3 ± 1.7
Combined	1.1 ± 1.3	1.7 ± 1.5	2.2 ± 1.8

Implant-level success rates based on marginal bone loss (mm) for the Astra Tech [A] and Brånemark [B] oral implant systems. Data are reported separately and combined, at baseline and at 1-, 3-, and 5-years post-placement. Values are expressed as means ± standard deviations. A *p*-value ≤ 0.05 was considered statistically significant and indicates a difference between [A] and [B]

**Fig. 4:**
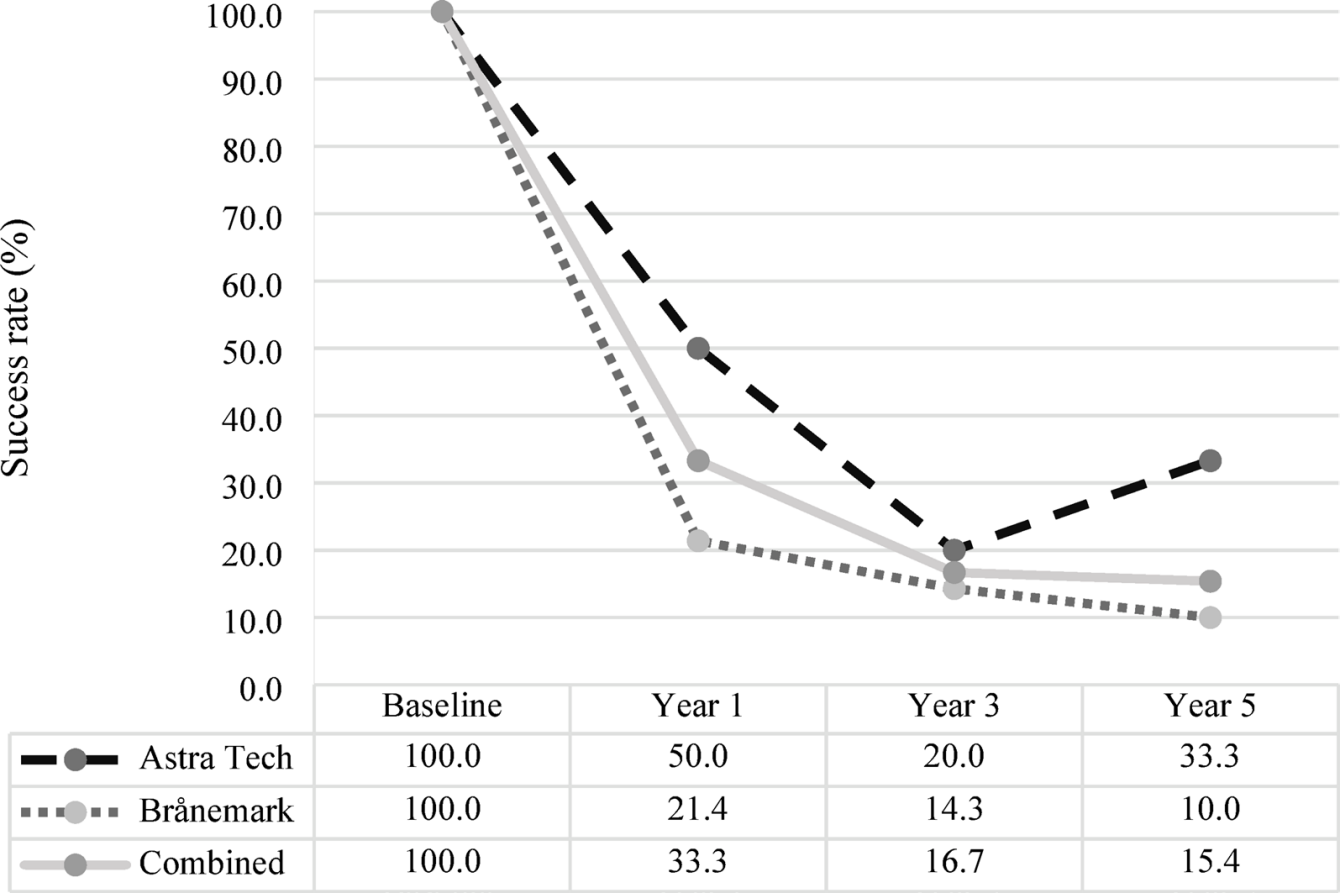
Patient-level success rates over time for Astra Tech and Brånemark implant systems. Patient-level success rates (%) for the Astra Tech [A] and Brånemark [B] oral implant systems, presented separately and combined, at baseline and at 1-, 3-, and 5-years following implant placement

In contrast, when assessing success at the patient-level at, 1-, 3-, and 5-years following implant placement, no statistically significant differences were observed between the two implant systems at any follow-up point. At 1 year after implant placement, 33% (8/24) of patients met the success criteria across both systems, with system-specific rates of [A = 5/10; 50%] versus [B = 3/14; 21%]. At the 3-year follow-up, 17% (4/24) of patients met the success criteria, with [A = 2/10; 20%] versus [B = 2/14; 14%]. By year 5, overall patient-level success had decreased to 15% (2/13), with [A = 1/3; 33%] versus [B = 1/10; 10%] (Fig. 4).

For the secondary endpoint (2b), i.e., the long-term survival-, and success rates of the two implant systems [A and B], 54% (14/26) of the original patient cohort were identified in the Swedish population register maintained by The Swedish Tax Agency. Of the remaining 46% (12/26), 11 patients were confirmed deceased and 1 had emigrated from Sweden. Among the patients identified in the register, 71% (10/14) were either unreachable or declined participation, resulting in 29% (4/14) being enrolled for the additional radiological examination. All digital panoramic radiographs obtained were of sufficient diagnostic quality for evaluation of the targeted variables. In total, 28 implants were assessed in these 4 patients, all of which belonged to the [B] implant system. The mean age at the time of implant placement was 61 years (range: 39–68 years). None of the patients had experienced implant loss at the time of the digital radiological follow-up, conducted approximately 30 years following the implant placement, corresponding to a long-term survival rate of 100%. With respect to long-term success, the mean marginal bone loss across all 28 implants was 2.6 ± 1.8 mm. However, due to the small sample size, no statical analysis was performed.

## Discussion

Despite advancements in implantology that have reduced the need for complex surgical procedures such as Le Fort I osteotomy with interpositional bone grafting, the academic and clinical relevance of evaluating implant survival and success following this approach remains significant. Although, previous studies have demonstrated favorable outcomes with oral implants in patients with severe maxillary atrophy, limited research has specifically focused on implants placed following this surgical technique. This gap in the litterature formed the rationale for the present study.

Historically, the use of interpositional bone grafting in conjunction with Le Fort I osteotomy has been a cornerstone technique for managing severe maxillary atrophy. However, it is important to interpret our findings within the context of contemporary treatment alternatives. Techniques such as zygomatic implants and short implants have gained popularity due to their graftless nature and reduced surgical morbidity. Zygomatic implants are increasingly used in cases of extreme bone resorption, offering high success rates and the potential for immediate loading. Nevertheless, these procedures are surgically demanding, associated with specific complications (e.g., sinusitis, soft tissue irritation), and typically require advanced surgical expertise. Short implants represent a less invasive option for patients with moderate atrophy, although their long-term survival in severely resorbed maxillae remains a subject of ongoing investigation [12–14]. In contrast, the Le Fort I osteotomy with interpositional grafting not only enables vertical and anteroposterior repositioning of the maxilla but also provides a predictable foundation for implant placement with favorable long-term outcomes, as demonstrated in our cohort.

Five years following implant placement, the implant-level survival rate for the two oral implant systems evaluated was 94.1%. These results are encouraging, particularly considering that advanced surgical procedures were required to achieve the adequate bone volume prior to placement. Notably, our findings align with those of a previous study in which severely atrophic maxillae were managed with autogenous bone grafts harvested from the iliac crest prior to implant placement [15]. Other studies have likewise reported favorable long-term outcomes for osseointegrated implants placed following Le Fort I osteotomy with interpositional bone grafting [4, 5].

When survival analysis was repeated at the patient-level, all implant losses, regardless of implant system, occurred within the first 3 years post-placement. Among the 26 patients, 6 experienced a total of 12 implants losses during this period. This variability may be attributed to individual patient differences, as most patients experienced no implant loss, while a few lost multiple implants. Notably, at the patient-level, the survival rate for the Brånemark system implants remained stable between years 2 and 3 (73.7%). In contrast, the implant-level survival rate for the same system declined slightly from 92.1% to 91.2% during the same period, reflecting multiple implant losses in a single patient. Although Astra Tech implants demonstrated a higher overall survival rate than the Brånemark system implants, the difference was not statistically significant. These findings are consistent with those reported in a previous study [9], although that study compared the two implant systems in the context of conventional implant surgery.

The combined implant-level success rates for the Astra Tech and Brånemark systems were 70.7%, 64.6%, and 56.1% at 1-, 3-, and 5-years post-placement, respectively. In a previous systematic review, of 23 longitudinal studies encompassing 7711 implants, reported success rates ranged from 35 to 100%, with an average follow-up of 13 years [16]. This wide variation is largely attributed to differing definitions of success employed across studies [11, 16]. Notably, approximately 50% of the studies, including the present one, adopted the success criteria proposed by Albrektsson [10]. It is important to acknowledge, however, that Albrektsson's criteria encompass clinical parameters that were not assessed in the present study due to the lack of available clinical data. This limitation significantly reduces the comprehensiveness of the success evaluation.

Astra Tech implants demonstrated significantly higher implant-level success rates than Brånemark implants at both 1-, and 3-years post-placement evaluation. This finding is consistent with previous research, which reported that, 1 year after placement, 29% of the Brånemark implants exhibited at least 1 implant with ≥ 2 mm of marginal bone loss, compared to only 16% of Astra Tech implants [9]. By the 5-year follow-up, the difference in success rates between the two systems was no longer statistically significant, although Astra Tech implants continued to show slightly higher success values.

The long-term outcomes of the two implant systems were evaluated approximately 30 years after implant placement, revealing a 100% survival rate and a mean marginal bone loss of 2.6 ± 1.8 mm. While these findings suggest sustained clinical performance over time, consistent with previous studies reporting high long-term survival rates and minimal bone resorption for oral implants [16–19], it is important to emphasize the significant limitations of this component of the study. Most notably, the long-term evaluation included only four patients and 28 implants, all of which belonged to the Brånemark system. This small and non-representative sample substantially limits the generalizability of the results and precludes meaningful statistical comparisons between implant systems. As such, the findings should be interpreted as descriptive and exploratory rather than conclusive.

Further limitations include the use of implant-level analyses, which may be affected by the potential correlation between multiple implants placed within the same patient. To address this issue, parallel analyses were conducted at both the implant and patient levels. The patient-level analysis, which accounts for the non-independence of implants within patients, yielded results consistent with those from the implant-level analysis. This consistency supports the validity of the implant-level findings. Another limitation is the absence of data on patient-related factors such as smoking status, systemic health conditions, history of periodontal disease, and bone quality, all of which are known to influence long-term implant outcomes. Moreover, although marginal bone levels were assessed using panoramic radiographs, which have been reported to demonstrate a high level of agreement with intraoral methods [20], they offer lower resolution compared to periapical radiographs, which are currently recommended for such evaluation [21]. In general, the panoramic radiographs were of sufficient diagnostic quality to determine the targeted variables. However, in some cases, evaluation of the marginal bone level on one or both implant surfaces (mesial and/or distal) was limited due to suboptimal patient positioning, including improper tongue or head placement, or superimposition of the cervical spine. While intraoral radiographs could have provided higher image quality, panoramic imaging was chosen to ensure consistency with previous timepoints and to enable standardized comparison across the entire study period.

## Conclusion

Le Fort I osteotomy with interpositional bone grafting, followed by full-arch implant placement, demonstrates favorable long-term survival-, and success rates. Both Astra Tech and Brånemark implant systems proved adequate for this treatment approach. Therefore, this technique remains a viable and clinically relevant option in selected cases where bone volume is insufficient, conventional implant therapy is not feasible, and the sagittal or vertical jaw relation is unfavourable.

## Data Availability

The datasets used and/or analyzed during the current study are available from the corresponding author on reasonable request.

## Abbreviations

[A]: Astra Tech implant system (used as an abbreviation within the study)
ALADA: As Low as Diagnostically Acceptable
ALARA: As Low as Reasonably Achievable
[B]: Brånemark implant system (used as an abbreviation within the study)
IDS7: Image and Documentation System version 7
SPSS: Statistical Package for the Social Sciences
WMA: World Medical Association
